# A Unique Case of a Right Atrial Myxoma Infected With Escherichia coli

**DOI:** 10.7759/cureus.25394

**Published:** 2022-05-27

**Authors:** Masi Javeed, Hanan Gruhonjic, Taylor Kirkman, Christos Pitarys, Rami Akel

**Affiliations:** 1 Internal Medicine, HCA Florida Bayonet Point Hospital, Hudson, USA; 2 Cardiology, HCA Florida Bayonet Point Hospital, Hudson, USA; 3 Interventional Cardiology, HCA Florida Bayonet Point Hospital, Hudson, USA

**Keywords:** escherichia, transthoracic echocardiogram, trans-esophageal echocardiogram, right atrial cardiac mass, right atrial myxoma

## Abstract

A 70-year-old white male, with past medical history of coronary artery disease, peripheral arterial disease status-post bilateral femoral artery stents, insulin-dependent diabetes mellitus, hypertension, hyperlipidemia, arthritis, tobacco use, and alcohol use, presented with shortness of breath and an abnormal finding on a recent transesophageal echo. This had revealed a large, fixed mass in the right atrium. Our differential diagnosis had included thrombus, endocarditis, myxoma, papillary fibroelastoma, sarcoma, and metastatic tumor. The cardiothoracic surgeon excised this mass, which upon culturing, revealed what we found to be the only reported case of an atrial myxoma infected with Escherichia coli. In addition to surgical excision, the patient was treated with six weeks of intravenous cefepime.

## Introduction

Atrial myxomas are the most common primary cardiac neoplasms and compose more than 50% of benign cardiac tumors [1]. Around 75% originate in the left atrium near the mitral annulus or the fossa ovalis border of the interatrial septum [2]. About 20% arise in the right atrium and 5% can originate from both the atria and ventricle [3]. Most often the tumors are pedunculated and gelatinous in texture and their diameter can range from 1-15 centimeters [1]. The surface texture can be friable, villous, or smooth, with the friable and villous masses more commonly producing embolic events [1]. Myxomas can also produce cytokines that create constitutional symptoms such as fever, malaise, fatigue, and anorexia [2]. Infected myxomas are especially rare, with infrequent reports in the literature [3].

## Case presentation

**Figure 1:**
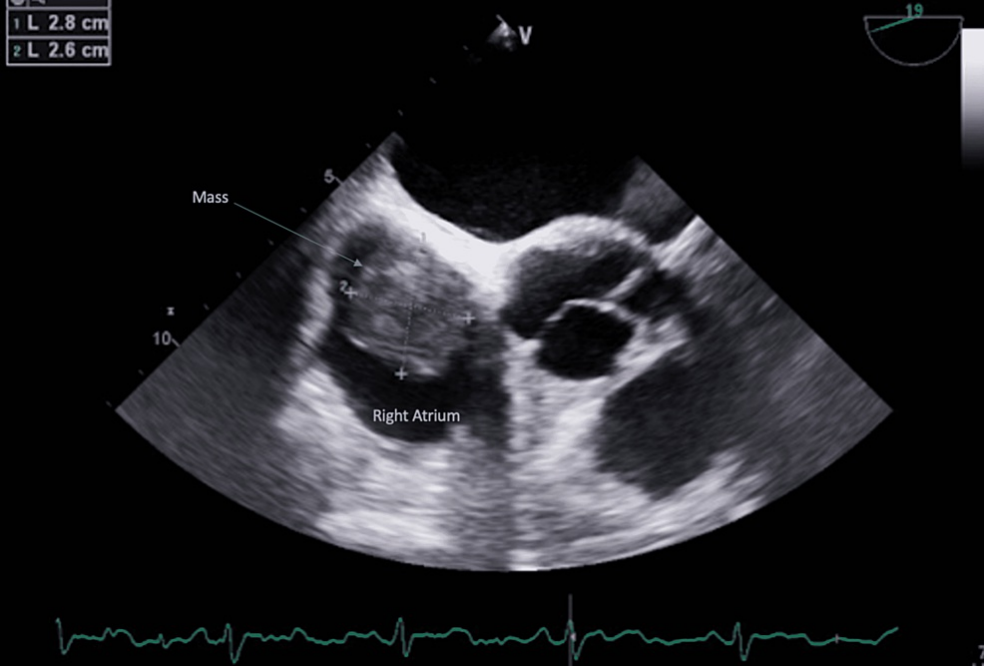
Mid-esophageal short axis view with omniplane angle of 19 degrees showing a 2.8 x 2.6 centimeter right atrial mass adjacent to the interatrial septum

A 70-year-old white male presented to the hospital with shortness of breath and a recent abnormal finding on transesophageal echocardiogram. The patient had experienced shortness of breath for four weeks prior to admission. He underwent labs, an electrocardiogram, and a chest x-ray in the outpatient setting; these were all unremarkable. However, a transthoracic echocardiogram revealed concern for a mass in the right atrium. This prompted a transesophageal echocardiogram to be ordered. The patient had this transesophageal echocardiogram done one week prior to admission which revealed a large, fixed mass with dimensions of 26 x 28 centimeters; ejection fraction was 50-55% (Figure 1).

**Figure 2:**
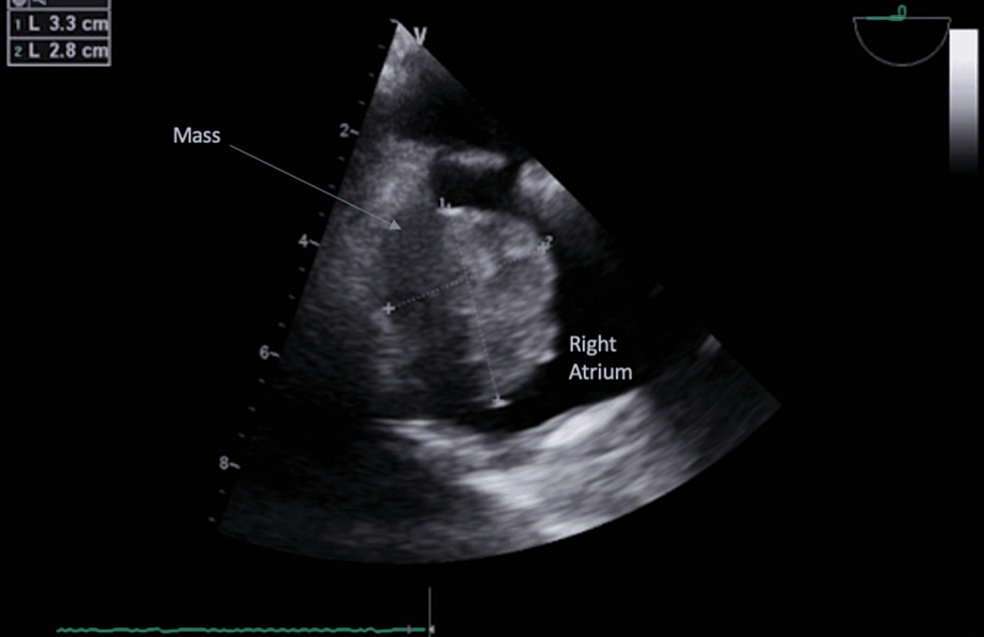
Mid-esophageal four chamber view with omniplane angle of 0 degrees showing a 3.3 x 2.8 centimeter right atrial mass adjacent to the interatrial septum

The patient was admitted to the hospital for possible excision of this right atrial mass. Initial vitals, physical exam, laboratory findings, and blood cultures were unremarkable. Electrocardiogram revealed normal sinus rhythm. Left heart catheterization and ventriculography were done for pre-op evaluation for excision of right atrial mass. This revealed the proximal first obtuse marginal artery to have a long 80% stenosis; ejection fraction was 65%. Therefore, the patient was accepted for surgery and was also planned for a single bypass graft to the first obtuse marginal artery. Cardiothoracic surgery was consulted. Although the Society of Thoracic Surgeons' risk score for coronary artery bypass graft surgery revealed 1.138% mortality and 11.269% morbidity and/or mortality, the patient was amenable and subsequently underwent coronary artery bypass graft surgery with the reversed segment of the right greater saphenous vein to the first obtuse marginal artery. Perioperative transesophageal echocardiogram revealed the mass again with dimensions of 3.3 x 2.8 centimeter on this occasion; the patient subsequently underwent excision of this right atrial mass along with atrial septal defect repair with autologous pericardium (Figure 2).

The mass was a firm, polypoid, yellow tumor greater than 3 centimeters arising from the atrial septum. This mass was sent to pathology as well as microbiology. Per pathology, the mass was found to have multiple hemorrhagic areas upon sectioning. On microscopic examination, the mass was composed of polygonal and stellate cells with irregular nuclei within myxoid stroma. Immunohistochemical stains as well as histochemical stains revealed the mass was CD34, calretinin, aclian blue, and mucicarmine positive. Immunophenotypic findings were consistent with atrial myxoma. Notably, per microbiology, E. coli was cultured from the mass. An infectious disease specialist was consulted. Of note, patient was determined to have poor dentition for the last two years and the patient was even supposed to have a procedure done for a dental abscess one year ago. The infectious disease specialist recommended six weeks of intravenous cefepime for treatment. Otherwise, the patient also experienced post-op atrial fibrillation with tachycardia-bradycardia syndrome. Since the onset was within the last 48 hours, transesophageal echocardiogram to assess the left atrial appendage was not deemed necessary. Therefore, direct current cardioversion was performed. Also, a permanent pacemaker was inserted instead of a temporary device because the pathologist had deemed the infected mass to have been completed excised, post-operative blood cultures were negative for more than 72 hours, and the infectious disease specialist was agreeable with the procedure. The rest of the patient’s hospitalization was unremarkable and he was discharged soon after. Upon follow-up, the patient had completed the course of antibiotics. He denied chest pain, paroxysmal nocturnal dyspnea, and orthopnea. No further complications were noted.

## Discussion

Infected myxomas are very rare with only 39 reported cases as recently as 2015 [3]. They are clinically significant because they are associated with higher incidence of fever, sepsis and septic shock, disseminated intravascular coagulation, and embolic events; this is largely due to increased fragility [4-6]. Embolization of an infected myxoma can lead to intracerebral ischemia, hemorrhage, lung or brain abscess, mitral or pulmonary valve endocarditis, and other downstream effects [5]. Diagnosis requires a myxoma documented by pathology with associated microorganisms on pathology or positive blood cultures [7]. Additionally, transesophageal echocardiogram has shown high diagnostic sensitivity approaching 100% [8]. The differential diagnosis for infected myxoma includes non-infected myxoma, mural endocarditis, and infected intracardiac thrombus. Risk factors for development of myxomal infection include recent dental procedures or poor dental health, recent infections, invasive procedures, intravenous drug use, and immunocompromised state [9].

In 1998, the most commonly isolated pathogens involved in myxomal infections were found to be streptococci, staphylococci, enterococci, Klebsiella, Neisseria, and Actinobacillus [7,8]. Subsequent reports have found species of Porphyromonas, Candida, Gemella, Histoplasma, and Kodamaea additionally implicated [5,7,8,10,11]. Prior to this case, to the best of our knowledge, there has been no documented case of a myxomal E. coli infection. It is additionally unique that the infected myxoma in this report occurred within the right atrium, deviating from the more common presentation within the left atrium [3]. 

With regards to management, surgical excision of the infected myxoma and intravenous antibiotics appear to be therapeutic [6]. Some reports note an urgency for surgical removal in order to prevent catastrophic embolization, as infected myxomas are two- to threefold more likely to embolize as compared to noninfected [12]. Other reports however opted to pretreat with antibiotics for several weeks prior to surgical removal [3]. In our patient, surgical excision of the E. coli-infected myxoma and six weeks of intravenous cefepime were curative.

## Conclusions

We conclude that there are only a few documented cases of infected atrial myxoma in the literature. There are even fewer cases where myxomas are infected with gram-negative organisms and none to our knowledge where they are infected with Escherichia.

Transesophageal echocardiogram, pathology, and microbiology are all useful tools in diagnosis. Urgent surgical excision and intravenous antibiotics are the preferred treatment for infected myxoma. However, further research could attempt to compare outcomes in cases in which pretreatment with antibiotics was followed by surgical excision versus cases in which surgical excision was followed by subsequent intravenous antibiotics.

## References

[1] Cohen R, Singh G, Mena D, Garcia CA, Loarte P, Mirrer B (2012). Atrial myxoma: a case presentation and review. Cardiol Res.

[2] Belgi Yıldırım A, Er A, Küçük M, Ozbilim G (2011). Infected giant left atrial myxoma: an unusual phenomenon. Anadolu Kardiyol Derg.

[3] Yuan SM (2015). Infected cardiac myxoma: an updated review. Braz J Cardiovasc Surg.

[4] Chan V, Veinot JP, Hynes M, Lapierre H, Ruel M (2007). Infected right ventricular myxoma and pulmonary valve endocarditis. J Thorac Cardiovasc Surg.

[5] Wang TD, Chang SC, Chiang IP, Luh KT, Lee YT (1996). Infected left atrial myxoma caused by Gemella morbillorum. Scand J Infect Dis.

[6] Yoshioka D, Takahashi T, Ishizaka T, Higuchi T (2011). Successful surgical resection of infected left atrial myxoma in a case complicated with disseminated intravascular coagulation and multiple cerebral infarctions: case report. J Cardiothorac Surg.

[7] Rogers E, Weyman A, Noble R (1978). Left atrial myxoma infected with histoplasma capsulatum. A J Med.

[8] Revankar SG, Clark RA (1998). Infected cardiac myxoma. Case report and literature review. Medicine (Baltimore).

[9] García-Quintana A, Martín-Lorenzo P, Suárez de Lezo J (2005). Infected left atrial myxoma. Rev Esp Cardiol.

[10] Jayaweera JA, Kothalawala M, Sooriyar S (2021). Infected tricuspid valve myxoma with Kodamaea ohmeri: case report. Indian J Med Microbiol.

[11] Joseph P, Himmelstein DU, Mahowald JM, Stullman WS (1980). Atrial myxoma infected with Candida: first survival. Chest.

[12] Trimeche B, Bouraoui H, Garbaa R, Mahdhaoui A, Ben Rhomdane M, Ernez-Hajri S, Jeridi G (2009). Systemic embolism and septic shock complicated left atrial myxoma: case report. Case Rep Med.

